# Enhanced Biomimetics of Three-Dimensional Osteosarcoma Models: A Scoping Review

**DOI:** 10.3390/cancers16010164

**Published:** 2023-12-28

**Authors:** Vinesh Sandhu, Deniz Bakkalci, Siyi Wei, Umber Cheema

**Affiliations:** 1 Division of Medicine, UCL Medical School, University College London (UCL), 74 Huntley Street, London WC1E 6DE, UK; zchav04@ucl.ac.uk; 2 UCL Centre for 3D Models of Health and Disease, Division of Surgery and Interventional Science, University College London (UCL), Charles Bell House, 43-45 Foley Street, London W1W 7TS, UK; siyi.wei.20@ucl.ac.uk

**Keywords:** osteosarcoma, tissue engineering, 3D model, biomimicry, drug screening, stromal cells, tumour microenvironment

## Abstract

**Simple Summary:**

This review explores various studies to understand how 3D cell cultures mimic real tumour conditions and respond to anticancer drugs. This study focuses on osteosarcoma (OS), which is a type of bone cancer. The findings of these studies revealed that 3D OS models, especially those with specific scaffolds, better replicate the OS tumour environment by influencing their growth and resistance to anticancer drugs. This review highlights the importance of improving 3D OS models to better represent the actual tumour environment and increase the accuracy of drug testing. Future work should explore innovative 3D models and materials for more effective advancement in osteosarcoma research, with the hope of translating to better patient care.

**Abstract:**

This scoping review evaluated 3D osteosarcoma (OS) models’ biomimicry, examining their ability to mimic the tumour microenvironment (TME) and their drug sensitivity. Adhering to PRISMA-ScR guidelines, the systematic search revealed 293 studies, with 70 selected for final analysis. Overall, 64% of 3D OS models were scaffold-based, compared to self-generated spheroid models. Scaffolds generated using native matrix were most common (42%) with collagen I/hydroxyapatite predominating. Both scaffold-based and scaffold-free models were used equally for drug screening. The sensitivity of cancer cells in 3D was reported to be lower than that of cells in 2D in ~90% of the drug screening studies. This correlates with the observed upregulation of drug resistance. OS cells cultured in extracellular matrix (ECM)-mimetic scaffolds and native biomaterials were more resistant than cells in 2D. Co-cultures of OS and stromal cells in 3D models enhanced osteogenic differentiation, ECM remodelling, mineralisation, and angiogenesis, suggesting that tumour–stroma crosstalk promotes disease progression. Seven studies demonstrated selective toxicity of chemotherapeutics towards OS cells while sparing stromal cells, providing useful evidence for developing biomimetic tumour–stroma models to test selective drug toxicity. In conclusion, this review highlights the need to enhance biomimicry in 3D OS models for TME recapitulation, especially in testing novel therapeutics. Future research should explore innovative 3D biomimetic models, biomaterials, and advancements in personalised medicine.

## 1. Introduction

Osteosarcoma (OS) is the most prevalent primary bone cancer, with the highest occurrence among children and adolescents [1]. OS arises from the mesenchymal stem cells (MSCs) of long bone and is typified by the deposition of immature osteoid matrix by tumour cells. Although the aetiology remains largely obscure, evidence indicates that OS is unstable genetically and possesses intricate karyotypes [2]. The existing treatment regimen of OS comprises neoadjuvant chemotherapy, comprising methotrexate, doxorubicin (adriamycin) and cisplatin (Platinol) (i.e., the MAP regime), followed by surgical excision and adjuvant chemotherapy. OS remains a highly aggressive tumour with incidence of lung metastasis, calling for the need to develop novel therapeutic strategies and biomarkers [3].

**Figure 1:**
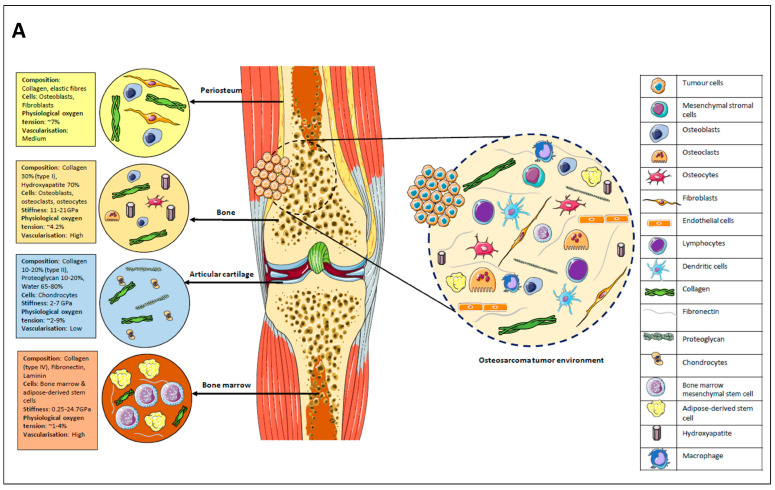

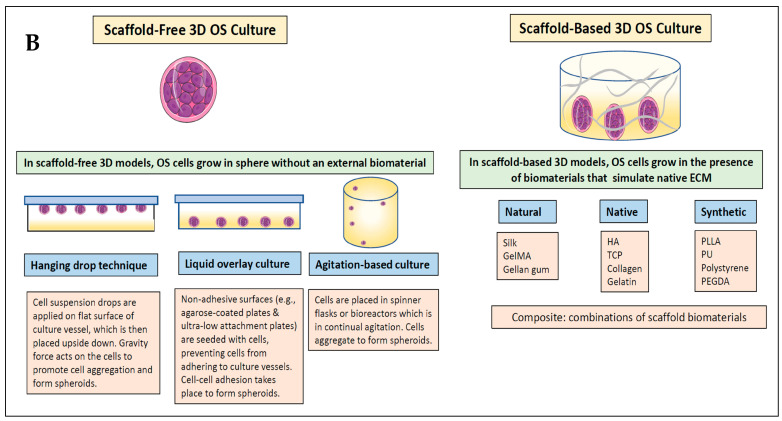
(**A**) The osteosarcoma tumour microenvironment (TME). This TME comprises bone and stromal cells (osteoblasts, osteocytes, osteoclast, chondrocytes, mesenchymal cells, and fibroblasts), endothelial cells, and immune cells (tumour macrophages, dendritic cells, and lymphocytes), and ECM comprises primarily hydroxyapatite and collagen. The 4 boxes (left of (**A**)) characterise the physiological parameters of a normal bone milieu. (**B**) Schematic representation of common 3D OS model cultures techniques. Both images were created with Servier Medical Art (Creative Commons Attribution 3.0 Unported Licence).

The OS tumour microenvironment (TME) is highly complex and includes mesenchymal stem/stromal cells, fibroblasts, osteoclasts, osteoblasts, osteocytes, haematopoietic cells, endothelial cells, immune cells (e.g., lymphocytes and macrophages), and adipocytes embedded in a mineralised extracellular matrix (ECM) [3,4] (Figure 1A). Evidence suggests that bone-marrow-derived mesenchymal stem cells (BMSCs) form a significant component of the OS microenvironment, with a strong tropism towards OS cells [5]. When they establish contact with OS cells, BMSCs differentiate into cancer-associated fibroblasts (CAF), releasing various cytokines that include interleukin (IL)-6 and IL-8 and monocyte chemoattractant protein-1 (MCP-1) in the TME [5]. This enhances OS aggressiveness in terms of invasiveness, motility, and trans-endothelial migration. Notably, MSCs’ existence in the TME favoured OS proliferation and metastasis, partly contributed to by c-c chemokine ligand (CCL)-5 produced by hMSC.

The optimal in vitro model should mimic the TME [3,4]. Two-dimensional in vitro culture does not recapitulate many in vivo tumour characteristics including tumour heterogeneity and tissue architecture. Compared to 2D monolayers, cancer cells in 3D models demonstrate variations in functional behaviours, cellular proliferation, and differentiation [6]. Gene/protein expression in 3D models of OS mimic a more precise depiction of the dynamic cell–cell and cell–ECM interactions in the TME. With a heightened understanding of the heterogeneity and complexity of TME, a plethora of novel 3D models have been designed by optimising the scaffold biomaterials and innovating 3D culture techniques [6]. Three-dimensional models can be classified into two broad categories: scaffold-based and scaffold-free (self-generating) models, as shown in Figure 1B.

Scaffold-dependent 3D models based on natural or synthetic materials can mimic the ECM and offer physical reinforcement for cell growth [6]. Factors considered when selecting 3D biomaterials include biocompatibility, bioactivity, longevity, biodegradability, biomechanics (elastic modulus and tensile strength), mass transport (physiological exchange of oxygen, nutrients, and soluble factors), and surface attachment [7,8]. While synthetic materials are stable and can recapitulate the mechanical stiffness of bone matrix, enhanced cell–matrix interactions are key features of natural materials [3]. 

Alternative techniques for 3D models include the seeding of cells to form self-generating spheroid-like structures without ECM (scaffold-free 3D cultures). These spheroids are constructed using various techniques (Figure 1B) including culture on low-adhesive plates, the hanging-drop approach, and agitation-based bioreactors and microfluidic devices. They exhibit the capacity to mimic tissue properties, showing dense cellular composition and good cellular crosstalk [9]. The 3D multicellular spheroids better represent the microenvironment, cellular heterogeneity, and phenotype of tumours and create an oxygen and nutrient gradient from the exterior to the spheroid core. These tumour cells aggregate into different cell zones—outer: proliferating cells, middle: nonproliferating quiescent cells, and inner core: necrotic cells, which mimic the in vivo tumour mass [8,9].

Biomimetic 3D OS models should resemble the TME in terms of cell growth, oncogenesis, and cell–cell/cell–ECM interaction to unravel the complexity of OS pathogenesis. These models can be used to help identify and discover novel molecular targets, anticancer drugs, and disease biomarkers, potentially translating into better therapeutics and diagnostics in bone cancer management. This is particularly potent given the recent FDA decision to remove the need for animal testing for a drug to be approved [10]. The FDA typically requires toxicity tests on animal models to check for drug toxicity. However, it has been argued that, even with this, 90% of drugs entering human clinical trials end up being unsafe and, worse, ineffective. A move towards the use of humanised 3D tissue models thus seems a logical way forward.

There is currently limited data on the biomimetics of 3D OS models and how the biomimicry of 3D OS models changes depending on the engineered features of these models. Therefore, to fill this gap, this review systematically analyses the literature on 3D OS models to explore the types and range of available evidence pertaining to their biomimicry and resultant use as informative models to predict response to drugs.

## 2. Materials and Methods

### 2.1. Search Strategy

This systematic search of the available literature was performed according to the Preferred Reporting Items for Systematic Reviews and Meta-Analysis Extension for Scoping Reviews (PRISMA-ScR) checklist [11]. Five electronic databases were used: PubMed, Ovid MEDLINE, Scopus, Web of Science (WoS), and Ovid Embase. The search was restricted to between January 2012 and December 2022 to screen papers from the last 10 years. The keywords and Medical Subject Headings (MeSH) terms used were “Osteosarcoma”, “3D model”, “three-dimensional model”, and “3d model”. The search was performed in combination with the Boolean operators (‘AND’ and ‘OR’).

### 2.2. Inclusion and Exclusion Criteria

Inclusion criteria included:

(a) Studies that included in vitro 3D cell culture models with explicit use of osteosarcoma cell lines (including humans, rodent, and canine species) and/or primary human cells;
(b) Peer-reviewed journal articles reporting original research (as stated in (a)), published in English.

Exclusion criteria included:

(a) Use of in vitro 3D models that did not use osteosarcoma cell lines;
(b) In vivo, in silico, xenografts, and two-dimensional (2D) experiments;
(c) Review articles, commentaries, editorials, conference abstracts, non-full text, and non-English publications.

### 2.3. Definitions

Scaffold materials were divided into natural, synthetic, native, or composite [4,12]. For this scoping review, native scaffolds were defined as any matrix component found in human tissues, and natural scaffolds were defined as materials found naturally but not within human tissues (e.g., silk and alginate), whereas synthetic scaffolds were synthesised materials. Composite scaffolds comprise a combination of biomaterials from at least two different scaffold groups (synthetic, natural, or native). Scaffold-free (self-generating) 3D models referred to OS cells grown without an external biomaterial for support.

### 2.4. Synthesising, Analysing, and Reporting Results

Data extracted from the studies were then collated, synthesised, and analysed. Charts, tables, and diagrams were used to present the findings. The data were displayed in a manner that was aligned with the research objectives, which included the following:

(1) Biomimetic scaffolds used for 3D OS models.
(2) Biomimetics of 3D OS models related to anticancer drug/therapy testing. This included comparing the differences in drug sensitivities between the 3D and 2D OS models and identifying any other biomimetic factors potentially influencing drug resistance.
(3) Biomimetic response of stromal cells in 3D OS models.

Because of the highly heterogenous experimental variables between studies (variations in osteosarcoma cell lines, 3D OS model, stromal cells, scaffold biomaterial, drug types, and methods used to determine drug effectiveness) and diversity of research focus among the studies, it was not possible nor feasible to perform a meta-analysis and statistical analysis of data. Therefore, this review provided both quantitative and qualitative assessments of the study findings. The Statistical Package for Social Sciences (SPSS, Chicago, IL, USA, Version 20.0) was used to create a database for quantitative data analysis. Original images were designed with Servier Medical Art software, licensed under a Creative Commons Attribution 3.0 Unported License.

## 3. Results

### 3.1. Flowchart for Study Selection

**Figure 2:**
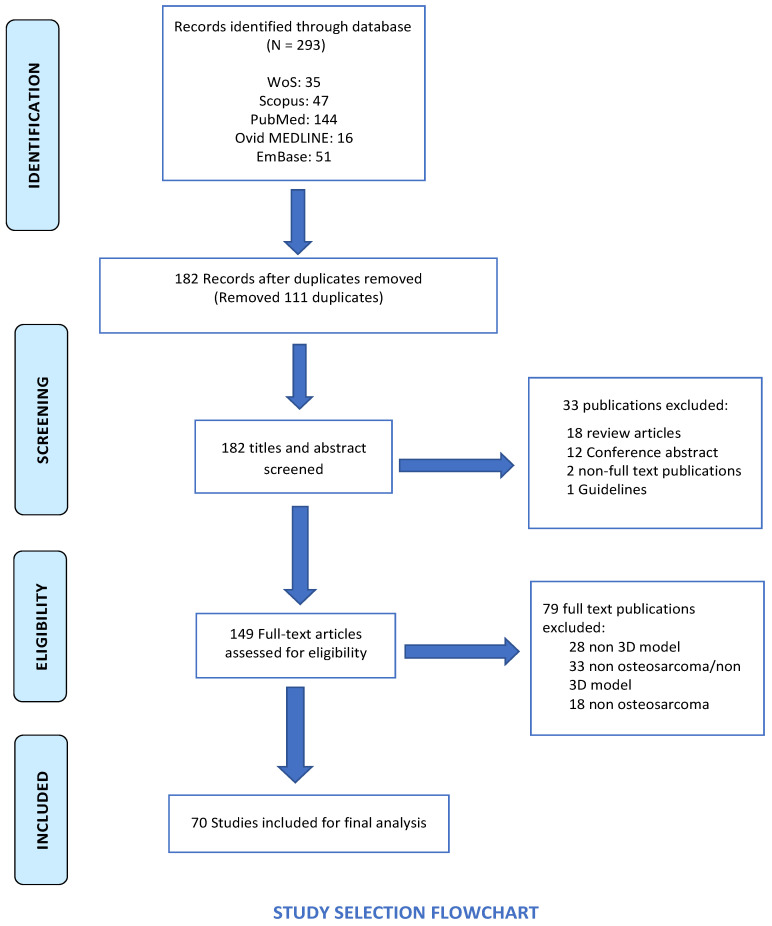
PRISMA study selection flowchart showing the total of publications removed at various stages and the reasons for the exclusions.

The search involving five electronic databases generated 293 potentially eligible studies, which were reduced to 182 after duplicate removal. Following title and abstract screening, 33 studies were excluded. The full texts of 149 studies were reviewed, from which a total of 79 studies were excluded: non-3D model (*n* = 28), non-OS/non-3D model (*n* = 33), and non-OS (*n* = 18). The full search is illustrated in the PRISMA flowchart (Figure 2)**.** Finally, 70 studies fulfilling the inclusion/exclusion criteria were included in this review [13,14,15,16,17,18,19,20,21,22,23,24,25,26,27,28,29,30,31,32,33,34,35,36,37,38,39,40,41,42,43,44,45,46,47,48,49,50,51,52,53,54,55,56,57,58,59,60,61,62,63,64,65,66,67,68,69,70,71,72,73,74,75,76,77,78,79,80,81,82]. Of these, 40 studies included drug screening, and 21 studies had incorporated stromal cells in the 3D OS model.

### 3.2. 3D Scaffolds Are Most Commonly Used to Engineer Osteosarcoma Models Compared to Self-Generated Spheroids

**Figure 3:**
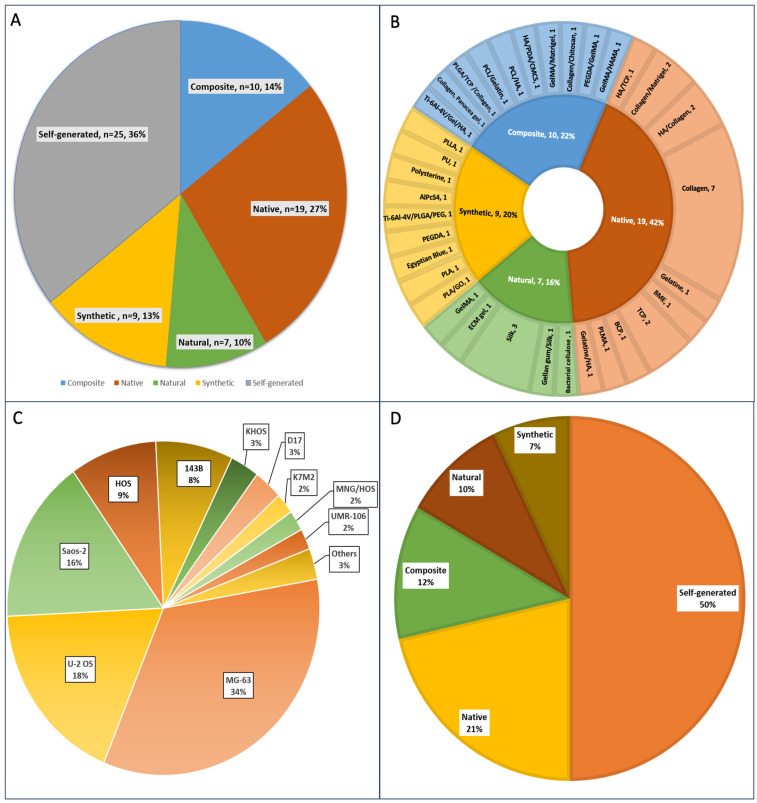
(**A**) Biomimetic scaffold-based and scaffold-free 3D OS culture models. Percentages represent the number of 3D models that are either scaffold-based or scaffold-free (self-generated) of the total 70 studies. (**B**) Types of biomimetic materials used in the 45 scaffold-based 3D OS models. The inner circle represents the percentage of the native, natural, synthetic, and composite scaffolds, while the outer circle shows the type of biomaterials used for each scaffold category. (**C**) The osteosarcoma cell lines used in 70 studies involving both scaffold-based and self-generated 3D OS models. All are human OS cell lines except D-17 (canine), K7M-2 (mouse), and UMR-106 (rat). (**D**) Types of scaffolds and non-scaffold (self-generated) 3D OS models used for drug testing. Percentages represent the number of 3D OS models that were either scaffold-based (using various biomaterials) or scaffold-free (self-generated) of the total of 40 drug-related studies.

Overall, scaffold-based models were the most common 3D OS model (64%, *n* = 45), while 36% (*n* = 25) were scaffold-free (self-generated) (Figure 3A). Native biomaterials mimicking the extracellular matrix of bone tissue were the most widely used, comprising 42% (*n* = 19) of the scaffold-based material (Figure 3B)**.** Amongst the native scaffolds, the most common biomaterial was collagen I (*n* = 11), followed by hydroxyapatite (HA) (*n* = 4) and tricalcium phosphate (TCP) (*n* = 3). Scaffolds blended with two or more different biomaterials to obtain composites were the second most common (22%, *n* = 10). Within the composite scaffolds, combinations of collagen, gelatine methacryloyl (GelMa), and HA (3 each) aimed at reproducing the biomimetic features of in vivo tumours, and these were the most frequently used. Synthetic scaffolds accounted for 20% of scaffolds used for OS models and these comprised a wide array of synthetic biomaterials, including polylactic acid (PLA) and polyethylene glycol diacrylate (PEGDA), used either individually or in combination. Amongst the natural polymers, silk predominated (*n* = 4), comprising 57% of the natural scaffold biomaterial. Overall, 31% (14/45) and 15.5% (7/45) of the scaffold-based 3D models used collagen and HA, respectively, making these the most widely used scaffold biomaterials.

In delineating the nuances between 3D spheroid models and osteosarcoma cells forming spheroids within 3D models, it is crucial to understand the distinction in their compositions and implications. A 3D spheroid model typically refers to a configuration where cells aggregate and self-assemble into a spherical structure. In this review, 3D OS spheroids were successfully formed in all 25 scaffold-free (self-generated) models. The predominant techniques for generating these spheroids were liquid overlay cultures, employing methods such as ultra-low attachment plates and agarose-coated plates (*n* = 18). The hanging drop method was utilised in five cases, while a microfluidic system was used in one instance.

Among the 45 3D scaffold-based models, osteosarcoma spheroids were present in 11 of them. Among these, nine cases [16,24,34,40,41,46,53,59,60] utilised pre-formed spheroids created through liquid overlay techniques (*n* = 6) and hanging drop methods (*n* = 3). These pre-formed spheroids were later embedded with the 3D scaffold. In the remaining two cases, spheroids were generated within the 3D scaffolds after seeding the scaffold with OS cells [27,52]. However, in the majority of cases (34 of 45), the OS cells were seeded and cultured within the 3D scaffold without demonstratable formation of spheroids.

A wide spectrum of OS cell lines was used to generate 3D cultures. MG-63 was the most used OS cell line (>30% of studies), followed by U-2 OS (18%) and Saos-2 (16%) (Figure 3C). Pierrevelcin et al. (2022) used OS patient-derived cells (PDCL) [41].

### 3.3. Three-Dimensional OS Models Are Utilised for Anticancer Drug/Therapy Screening

In 40 of the 70 studies, the 3D OS models developed were used as a platform for testing chemotherapeutic drugs, with equal use of self-generated (*n* = 21) and scaffold-based models (*n* = 21), as shown in Figure 3D. Amongst the scaffold-based models used for drug testing, the native scaffold was the most common (9/21, 43%), followed by the composite scaffold (5/21, 24%). The distribution of OS cells used for drug testing paralleled the overall OS cells used, with MG-63 (33%) and U-2 OS (22%) predominating.

**Figure 4:**
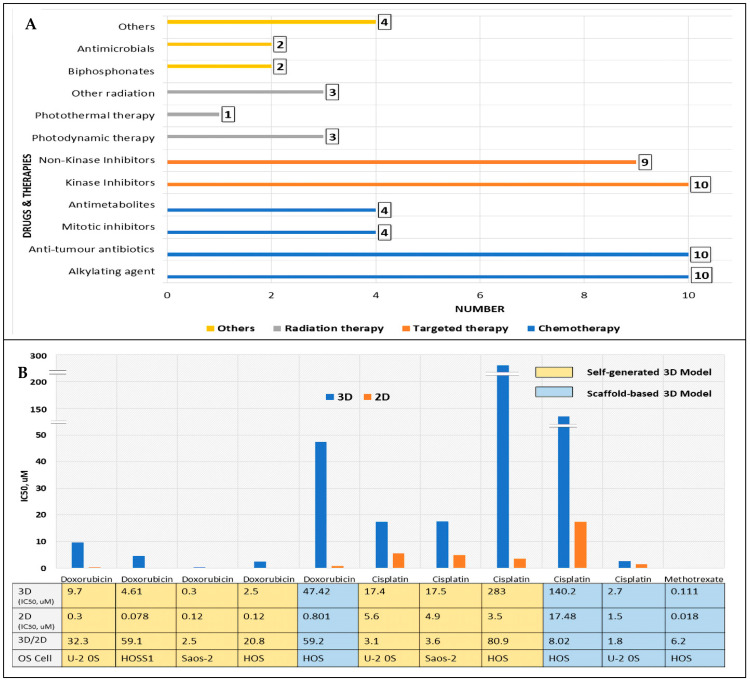

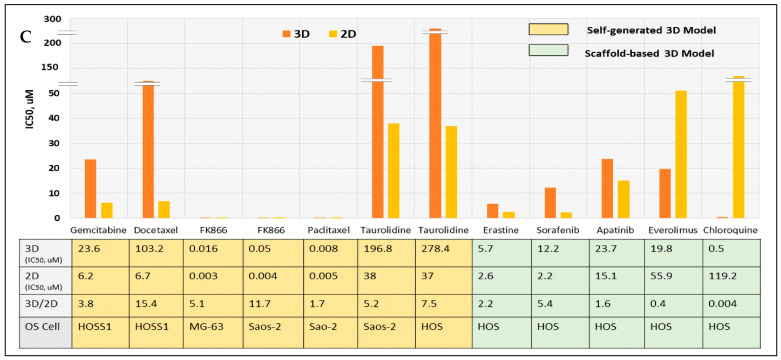
(**A**) Types of drugs/therapies used in drug testing studies, comprising various classes of chemotherapy, targeted therapy, radiation therapy, and other drugs. (**B**) Comparison of IC_50_ and the ratio between 3D and 2D for methotrexate, doxorubicin, and cisplatin (MAP) drugs. (**C**) Comparison of IC_50_ and the ratio between 3D and 2D for drugs other than the MAP drugs. Details of various OS cell lines and types of 3D OS models used (scaffold-based vs. scaffold-free) are included [13,14,22,25,28,29,58].

As shown in Figure 4A, chemotherapy drugs were the most common drugs tested; 28 chemotherapy drugs were tested in 40 studies. Targeted therapy comprising various kinase and non-kinase inhibitors formed the second largest group (*n* = 19). In seven studies, radiation therapy was used, the most common being photodynamic therapy (*n* = 3). Individually, the most common drugs tested were cisplatin (*n* = 9) and doxorubicin (*n* = 8), used either as monotherapy or in combination with other drugs.

### 3.4. Increased Drug Resistance in Biomimetic 3D OS Models: 3D vs. 2D Comparison of Drug Sensitivity Based on IC_50_

Of the 37.5% (15 of 40) of studies that explored the differences in drug sensitivities between 3D and 2D, 13 (of 15) studies demonstrated decreased drug sensitivity in 3D compared to 2D cultures. In 9 of these studies, this was based on IC_50_ comparison and the IC_50_ values are plotted in Figure 4B,C. However, for 2 (of these 9) studies, the plotting of IC_50_ values was not possible because the absolute IC_50_ value for either 3D/2D or both was not specified.

The IC_50_ was higher for 3D compared to 2D, suggesting higher drug resistance when tested in 3D OS cell cultures. Generally, the OS cells reacted differently to different anticancer drugs in various 3D OS cell culture models, with no specific trend observed. For the MAP drugs (Figure 4B), the 3D cultures’ IC_50_ values were between 2.5- and 59.2-fold higher for doxorubicin, between 1.8- and 80.9-fold higher for cisplatin, and 6.2-fold higher for methotrexate, compared to 2D monolayers. Meanwhile, for drugs other than MAP (Figure 4C), the IC_50_ values for 3D cultures ranged between 1.6- and 15.4-fold higher compared to the 2D monolayer. However, the ratios were reversed for everolimus and chloroquine (two autophagy-related drugs), with 2D cultures showing higher resistance compared to 3D cultures. Table 1 illustrates studies that showed decreased sensitivity in 3D compared to 2D, which were based on apoptosis comparison and relative cell viability OS cells.

Data were variable between studies due to differences in osteosarcoma cell lines, anticancer drugs/therapies used, methods used to determine drug effectiveness, and 3D OS models used, making inter-study comparison difficult. However, the individual studies allowed intra-study comparison as they were performed under standardised conditions. Osteosarcoma cells were more resistant to anticancer drugs in the presence of a native ECM component [24,40] and in the presence of a complex matrix incorporating matrix components of the OS niche [27]. Furthermore, increasing osteomimetic native material such as HA results in higher drug resistance [69].

### 3.5. Introducing Stromal Cells Can Induce Selective Anticancer Drug/Therapy Toxicity and Impact Other Biomimetic Responses

The most common stromal cells utilised were osteoblasts (*n* = 8) and fibroblasts (*n* = 6). The others included MSCs (*n* = 3), two each of adipose-derived stem cells (ASCs), bone marrow mesenchymal stem cells (BMSCs), and endothelial cells, while one study included macrophages. Amongst these, three studies included co-cultures of two stromal cells. In seven studies, stromal cells were used to demonstrate selective toxicity of anticancer therapy towards osteosarcoma cells while sparing the normal stromal cells (Table 2).

For example, Dobos et al. (2019) showed that 3D cultures of adipose-derived stem cells (ASCs) with OS spheroids showed no damage to healthy cells after TPE-PDT, showing precision of irradiation [34]. Moreover, Komez et al. (2020) showed that doxorubicin impact was explicitly evident on OS cells and not on healthy bone cells, human foetal osteoblast cells (hFOB), and human umbilical vein endothelial cells (HUVECs) [37]. A different study showed that crocin and bicarbonate showed selective cytotoxicity towards OS cells without affecting human foetal osteoblast cells (hFOB) [51]. Five studies demonstrated that stromal cells including fibroblasts, BMSCs, hMSCs, and osteoblasts augmented osteogenic differentiation and ECM formation, creating a favourable biomimetic ECM for OS cells [45,48,49,50,55].

Compartmentalised culture models were developed in two studies to enhance OS-healthy cell crosstalk to closely mimic the natural tumour microenvironment. Komez et al. (2020) developed a 3D model with two compartments: (1) a bone compartment composed of PLGA/TCL sponges seeded with hFOB and HUVECs and (2) a tumour compartment composed of collagen sponge seeded with Saos-2 OS cells [37]. This model investigated tumour–bone crosstalk by assessing HUVEC migration from normal bone to tumour compartment via showing increased angiogenic vascular endothelial growth factor (VEGF), fibroblast GF, and IL-8 within the tumour component. Second, Kundu et al. (2019) had tumour and bone compartments made from gellan gum–silk fibroin sponges that are seeded with OS cells and ASCs. The gellan gum–silk ratio of 3:1, with a compressive modulus of approximately 0.6 kPa, creates a tissue-mimicking microniche conducive for co-culturing OS and ASC, closely resembling the in vivo solid tumour-like structure of osteosarcoma [52].

In four studies, OS cells modified the surrounding stromal cells and this synergistic interaction increased tumour growth and invasion [35,40,44,46]. MSCs reacted to tumour-derived acidosis by activating an inflammatory cascade, promoting tumour invasiveness and metastasis [46]. Monteiro et al. (2021) showed that OS cells chemotactically attracted osteoblasts and MSCs, promoting tumour growth and invasion [40]. Three-dimensional co-culturing of OS-stromal cells (fibroblasts with human umbilical vein endothelial cells (HUVECs)) impacted angiogenic factors expression (IL-8 and VEGF-A) [44]. Furthermore, Jiang et al. (2020) found that OS cells were dependent on ECM stiffness, affecting the growth and progression of OS cells, while osteoblasts were mainly affected by ECM adhesion ligands [35].

## 4. Discussion

Biomimetic tissue models of osteosarcoma should mimic the TME in terms of cellularity, biophysical features, and biochemical gradients. These features can be introduced through the inclusion of physiologically relevant scaffolds and cells, using defined media. This work reviewed the use of biomaterials, as well as a cell-generated matrix to form osteosarcoma spheroids, to assess how 3D models affect osteosarcoma growth and response to chemotherapy. Overall, the use of native scaffolds was predominant, where collagen, HA, and TCP were most used. Native scaffolds are better able to recapitulate the main organic and inorganic bone elements, consisting of type 1 collagen and HA, respectively. HA and TCP (both comprising calcium/phosphate) are widely used in bone research owing to their excellent biocompatibility, chemical resemblance to native bone mineral, and capacity to promote the attachment, migration, proliferation, and differentiation of bone cells [32,51,59]. Three-dimensional collagen-based models were used to elicit OS invasiveness [27,28], therapeutic responses [27,28,60], and better mimicry of ECM for osteoblastic cultivation [63]. Collagen demonstrates excellent biodegradability and biocompatibility properties [83]. However, since pure-collagen hydrogels have poor mechanical strength, collagen-based composites are widely used to mimic bone ECM [83]. In this review, collagen/HA scaffolds [32] demonstrated high stability and porosity and comparable gene expression to native bone, while collagen/polylactic-co-glycolic acid (PLGA)/TCP [37] and chitosan/collagen mimicked the complex bone matrix [41].

A range of natural (GelMA, silk, and gellan gum) and synthetic (PEGDA, PLA, and graphene oxide (GO)) scaffolds were widely used for 3D OS model generation. GelMA was reported to support tumour stemness [81], cell adhesion, proliferation, and differentiation [22], while silk fibroin reportedly enhanced OS cell proliferation and attachment [77]. Synthetic biomaterials allow for accurate adjustment of material properties, including porosity, stiffness, and chemical composition. For example, Wang et al. (2022) generated a 3D PLLA scaffold mimicking native bone by modulating stiffness and porosity [75].

Natural scaffolds support bioactivity, biodegradability, biocompatibility, and enhanced cell–matrix interaction but have poor tensile strength [4,8,84]. Conversely, synthetic biomaterials have high tensile strength and elastic modulus but lack native moieties for cell attachment and, thus, biocompatibility [84]. Therefore, composites of natural/synthetic biomaterials were widely used to exploit the multiple favourable properties of biomaterials for enhanced OS growth. Adjusting gellan gum/silk composition yielded optimum stiffness for OS spheroid formation, suggesting the mechanical properties of hydrogels impact spheroid generation [52]. PLA/GO composites promoted biocompatibility, tensile strength, mineralisation, and OS cell proliferation more effectively than a pure PLA scaffold, providing a promising scaffold for osteogenesis [79]. Similarly, Porta et al. (2020) [71] demonstrated that HA/PCL composites enhanced mineralisation by U-2 OS cells, while a PEGDA/GelMA 3D model with increased matrix stiffness led to improved OS cell proliferation [35]. OS spheroids embedded in a GelMa/Matrigel scaffold promoted tumour invasion [24], while the Ti-6Al-4V/Gel/HA scaffold presented biocompatibility and osteointegration properties [48].

In this review, 3D OS models were demonstrably more resistant to both MAP and non-MAP drugs compared to 2D monolayers. This indicated that biomimetic 3D cultures might better recapitulate the complex in vivo microenvironment for drug screening. The differences might stem from the effects of 3D models on cellular proliferation, cell-cell/cell–ECM interactions, and gene/protein profile expressions [6]. Varied gene expressions between osteosarcoma cells grown in 3D compared to 2D models potentially contribute to drug sensitivity differences including upregulation of the ATP-binding cassette transport genes (ABC-B1, -C1, and -G2) implicated in drug efflux [13], multiple drug resistance protein 1 (MDR-1) [69].

The tissue-like architecture that 3D models provide alters the drug sensitivity of cells. The 3D spheroids reduce cancer cells’ accessibility to drugs compared to monolayer cultures. The hypoxic core augments drug resistance by promoting cell-cycle arrest at the GI phase [26,85]. Avnet et al. (2017) showed that the acidic OS microenvironment activates reactive MSCs, which secrete various chemotactic, pro-migratory, and pro-clonogenic factors through the NF-κB pathway, thereby promoting tumour migration, stemness, and resistance to chemotherapy. This underscores the importance of the stromal cells in chemotherapy resistance in OS, suggesting that targeting MSC holds promise for anti-cancer therapy [86]. Chemoresistance may be enhanced by adhesion-rich phenotypes, reducing drug penetration. Bai et al. showed that enhanced expression of ECM genes/proteins (e.g., COL1A1) and adhesion/gap junction molecules (connexin-26, -43, and -45) may facilitate drug resistance in 3D OS cultures [13].

Several studies in this review showed that adding biomimetic scaffolds or a native matrix enhanced drug resistance, compared to scaffold-free spheroids. Using a human methacryloyl platelet lysates (PLMA) scaffold, 3D OS cells displayed higher resistance to doxorubicin in the presence of a biomimetic ECM matrix [40]. Monteiro et al. (2020) showed that OS cells in spheroid-laden ECM-mimetic scaffolds were more resistant to lorlatinib compared to cells in scaffold-free spheroids [24]. Similarly, Pavlou et al. demonstrated that osteomimetic ECM conferred higher resistance compared to basic ECM [27]. Although widely used for drug testing, scaffold-free spheroids do not precisely mimic the natural TME, due to a lack of ECM components. The addition of biomimetic scaffolds enhances TME biomimicry [87]. ECM may impact drug effectiveness by changing the expression of drug targets, modifying drug availability, and altering intrinsic cell protective mechanisms, e.g., avoiding apoptosis [87].

Co-cultures of OS and resident stroma cells help explore the complexities of the interplay between these cells and ECM in cancer progression, invasion, and drug resistance. Stromal cells facilitate ECM remodelling, neoangiogenesis, and tumour cell migration and invasion by releasing diverse cytokines and growth factors. Segregating stromal and tumour compartments and then uniting them better mimics the natural tumour growth [88].

Tumour metabolism potentially impacts the OS–stromal cell crosstalk. In a 3D microfluid model, the MSCs reacted to OS-derived acidosis by triggering an inflammatory response, promoting OS cell invasiveness/metastasis [46]. TME displays regions of acidity, contributed to by uncontrolled cell proliferation and inadequate perfusion. Growing evidence suggests that an acidic environment stimulates epithelial-to-mesenchymal transition and ECM degradation and equips malignant cells with enhanced proliferative and invasive capacity [89]. 

This scoping review had some limitations. Studies retrieved were published in English only from January 2012 to December 2022. Thus, pertinent studies published in other languages and beyond the stated time frame may have been excluded. Due to highly heterogeneous data (variations in OS and stromal cells, 3D models, scaffold biomaterials, drugs, and techniques to evaluate drug effectiveness), statistical comparison was not feasible; thus, herein is presented quantitative and descriptive evidence of the data available. Additionally, only peer-reviewed studies were considered without evaluating grey literature evidence.

## 5. Conclusions and Future Work

This review provides an in-depth understanding of the various biomimetic aspects of 3D OS models, focusing on biomimetic scaffolds, 3D OS models for anticancer drug screening, and biomimetic impacts of stromal cells. Advancements have been made in 3D OS models to enhance their physiological relevance, biomimetic properties, and complexity by recapitulating the biophysical microenvironment and co-culturing OS/resident stromal cells. Collectively, this contributes towards a better understanding of OS carcinogenesis and anticancer therapies. To further mimic TME and promote tumour growth and metastasis, strategies such as tumour–stromal cell compartmentalisation have been employed. The findings from this scoping review provide useful insight into the potential application of biomimetic 3D OS models and the use of innovative techniques in 3D OS model engineering, paving the way for potential future research.

There is still a need for 3D OS models to better mimic the cellular interactions that occur in OS TME in vivo. This comprises leveraging the use of innovative biomaterials and advanced culture techniques, such as 3D bioprinting, advanced spheroids and organoids techniques, and microfluid-based 3D models to mimic OS TME. The emerging applicability of 3D bioprinting represents a notable advancement in tissue engineering and bone cancer research, as it can replicate the architecture, composition, and physico-mechanical attributes of bone [90]. Furthermore, it enables simultaneous deposition of the desired cell types such as stromal cells, immune cells, and CAFs. These OS cells can be incorporated into printable bioinks such as alginate, gelatin, GelMA, silk fibroin, chitosan, and collagen [90,91].

Furthermore, co-culturing of OS and stromal cells (e.g., MSCs, osteoblasts, endothelial, immune, and adipose cells) and adding compartments in the presence of biomimetic ECM further recapitulate the complexity of OS. In a biomimetic 3D colorectal cancer model, CAFs were found to enhance cancer invasion and angiogenesis [92]. However, the interaction between OS cells and CAFs is not well understood, emphasising the need to include CAFs in 3D OS models. As many OS deaths relate to lung metastases, incorporating tumour vasculature in 3D models is vital. Microfluidic techniques can be adjusted for culture under dynamic conditions to mimic in vivo blood flow and tumour invasion/metastasis [87].

To grasp the complexity of the OS TME, exploring its heterogeneity is essential, and single-cell RNA sequencing can serve as a promising tool to unveil valuable insights into cellular diversity within OS TME [93]. This contributes to the exploration of cellular mechanisms, cancer evolution, and the detection of drug-resistant cancer cell population, facilitating the development of therapeutic targeting strategies [93,94]. Considering the intrinsic heterogeneity and diverse subtypes of osteosarcoma [95], patient-derived 3D OS models offer a potential biomimetic model, capturing the diversity of this tumour. This review revealed that the use of patient-derived primary cells in 3D OS models was lacking. Thus, incorporating patient-derived cells in 3D OS models needs to be explored, as this fosters personalised medicine [87]. Furthermore, there is a scarcity of data linking 3D OS drug sensitivities and clinical responses amongst patients with osteosarcoma; further research could be conducted to explore this field. Finally, the broad and diverse range of findings gathered from this scoping review could serve as a starting point for subsequent systematic reviews targeting specific domains. Thus, the future work of 3D OS models should involve the creation of biomimetic models that could more accurately predict in vivo clinical outcomes, along with innovations in biomaterials and personalised medicine.

To sum up, the construction of an optimal 3D OS model hinges on replicating physiological conditions. To achieve this, a comprehensive model should incorporate various cell types, including stromal cells like CAFs and MSCs, in addition to OS cells. This ensures a faithful representation of the cellular diversity within the TME. There are 3D models where OS cells have been seeded into scaffolds, and sometimes, the OS cells will not necessarily form spheroids. Furthermore, the inclusion of endothelial cells is essential to emulate the intricate endothelial network and angiogenesis observed in osteosarcomas. The choice of 3D model type should prioritise native ECM components such as collagen and HA. A crucial, sometimes underestimated element is the faithful representation of the bone environment. Achieving bone-like structure through mineralisation and, when possible, bone nodule formation is paramount for enhanced physiological relevance. Incorporating bioreactor systems that mimic blood flow becomes pivotal, ensuring the model’s dynamic nature and elevating its physiological fidelity. Notably, achieving an optimum 3D osteosarcoma model necessitates a meticulous balance of these components to faithfully capture the intricacies of the TME and bone characteristics.

## Figures and Tables

**Table 1:** Studies showing higher resistance in 3D compared to 2D OS models (based on methods other than IC_50_ determination).

**Author** **[Ref.]**	**OS Cell**	**Drugs**	**2D** **(Apoptosis)**	**3D** **(Apoptosis)**	**3D OS Model**	**Comments**
**Based on apoptosis of 3D OS models vs. 2D models (1 study)**						
He J, 2022 [81]	K7M2 MG-63	Doxorubicin	21.6% 43.1%	3.14% 23.9%	Scaffold-based (GelMA)	Early apoptosis was lower for 3D compared to 2D culture. mRNA expression of BCL-2 (anti-apoptotic) in 3D culture was 3.22x higher than 2D culture.
**Author**	**OS Cell**	**Drugs**	**Comparison Relative to**		**3D OS Model**	**Comments**
**Based on relative cell viability comparison (3 studies)**						
Ohya S, 2021 [56]	MG-63	Paclitaxel Doxorubicin Cisplatin PAX (KCa1.1 inhibitor)	Relative to untreated OS cells		Self-generated	3D culture showed greater resistance to paclitaxel, doxorubicin, and cisplatin compared to 2D culture. KCa1.1 activity (conferring resistance) was higher in 3D than in 2D. KCa1.1 inhibitor, PAX, improved sensitivity to drugs.
Tan PH, 2013 [30]	143.98.2 Saos-2 U-2 OS	Doxorubicin	Relative to untreated OS cells		Scaffold-based (Silk)	3D cultures demonstrated decrease sensitivity to cisplatin (10-fold difference in IC_50_) and doxorubicin (100-fold difference) compared to 2D cells.
	143.98.2 Saos-2 U-2 OS	Cisplatin				
Tornin J, 2021 [32]	MG-63	PAR	Relative to untreated OS cells		Scaffold-based (Collagen/HA)	PAR (cold plasma-activated Ringer’s) solution reduced cell viability in a dose-response manner in 2D monolayer, while it enhanced proliferation in 3D cultures at the same increasing treatment times except at 240 s, which eliminated cells in both 2D and 3D cultures.

**Table 2:** Stromal cells used to show selectivity of anticancer therapy towards osteosarcoma cells in various 3D OS models using different anticancer drugs/therapy.

Author	Stromal Cells	OS Cells	3D Model	Drug	Comments
Dobos A, 2019 [34]	Adipose-derived stem cell (ASC)	MG-63	Scaffold-based (Gelatine)	Two-photon excited Photodynamic Therapy (TPE-PDT)	3D culture of ASC with OS spheroids showed no damage to healthy cells after TPE-PDT, showing precision of irradiation.
Komez A, 2020 [37]	Human foetal osteoblast cells (hFOB) and human umbilical vein endothelial cells (HUVECs).	Saos-2	Scaffold-based (PLGA/TCP/Collagen)	Doxorubicin	Doxorubicin impact was explicitly evident on OS cells and not on healthy bone cells, human foetal osteoblast cells (hFOB), and human umbilical vein endothelial cells (HUVECs).
Koski C, 2020 [51]	Human foetal osteoblast cells (hFOB)	MG-63	Scaffold-based (TCP)	Bicarbonate and Crocin	Crocin and bicarbonate showed selective cytotoxicity towards OS cells without affecting osteoblasts.
Li Volsi A, 2017 [38]	Human dermal fibroblast	U-2 OS	Scaffold-based (ECM gel)	Nutlin-3 loaded in nanorod	Nutlin-3 loaded in a nanorod system was more effective against OS cells compared to fibroblasts, while free nutlin-3 did not distinguish cancer from normal cells.
Sarkar N, 2020 [43]	Human foetal osteoblast cell (hFOB)	MG-63	Scaffold-based (TCP)	Soy isoflavones (genistein, daidzein, and glycitein)	Isoflavones showed no toxicity (with improved proliferation, viability, and differentiation) in osteoblast cells while reducing OS cell viability and proliferation.
Wu VM, 2017 [33]	Fibroblasts	K7M2	Self-generated	NP loaded with bisphosphonate and JQ1	The NP reduced the viability of OS cells more than fibroblast cells.
Marshall SK, 2022 [39]	Fibroblast (EGFR-)	MG-63 (EGFR+)	Self-generated	Doxorubicin and Na^131^I nanoparticle (DIE- NP)	The anti-EGFR NP achieved 80-fold higher efficacy in targeting the OS cells compared to fibroblast cells.

## Data Availability

All data generated or analysed during this study are included in this published article.
